# Immunity to gastrointestinal nematodes: mechanisms and myths

**DOI:** 10.1111/imr.12188

**Published:** 2014-06-19

**Authors:** Richard K Grencis, Neil E Humphreys, Allison J Bancroft

**Affiliations:** 1 Faculty of Life Sciences, University of ManchesterManchester, UK

**Keywords:** parasitic helminth, cytokines, inflammation

## Abstract

Immune responses to gastrointestinal nematodes have been studied extensively for over 80 years and intensively investigated over the last 30–40 years. The use of laboratory models has led to the discovery of new mechanisms of protective immunity and made major contributions to our fundamental understanding of both innate and adaptive responses. In addition to host protection, it is clear that immunoregulatory processes are common in infected individuals and resistance often operates alongside modulation of immunity. This review aims to discuss the recent discoveries in both host protection and immunoregulation against gastrointestinal nematodes, placing the data in context of the specific life cycles imposed by the different parasites studied and the future challenges of considering the mucosal/immune axis to encompass host, parasite, and microbiome in its widest sense.

This article is part of a series of reviews covering Mucosal Immunity appearing in Volume 260 of *Immunological Reviews*.

## Introduction

Infection by gastrointestinal (GI) nematodes is the norm, and almost any animal with a GI tract will have at least one species of GI nematode that infects it for at least part, but more likely, most of its lifetime. This also includes man in areas of the world without access to modern intervention strategies and accounts for over 1 billion infected individuals worldwide. These parasitic species conform to the standard general nematode morphology and undergo the characteristic four molts during growth and development. The parasitic species considered here are dioecious and life spans vary from weeks to years. Investigation of these infections is not only of direct relevance to human and animal health, but also because they present a constant and major challenge to the immune system especially via the intestinal tract. They have done so throughout the course of evolution and are likely to have been a major driving force in the evolution and development of immune responses in the GI tract (and at other mucosal surfaces). Thus, analysis of immune responses to such parasites has application not only to the development of diagnostics and approaches to immunological based control measures such as vaccination, but also to our fundamental understanding of immunological mechanisms *per se*.

Ultimately, to fully understand the immune response to these parasites, it is important to appreciate their epidemiology and life cycle strategies to provide a basis for interpretation of immunological analysis of studies undertaken in endemic regions where infection is acquired naturally. This will place laboratory studies in context and allow recognition of the limitations of each approach. Notwithstanding this, it is clear that study of immunity to GI nematodes has greatly enriched our overall understanding of host protective responses and generated new concepts of immune regulation.

## Natural history of GI nematode infection

Infection tends to arise from infective stages from the external environment or ‘soil’, and therefore, the major infective GI nematode species are also commonly known as soil-transmitted helminths (STH). For the most part, the data suggest that infections tend to be chronic/long lived in nature with individuals harboring infection for the majority of their lives. Thus prevalence of infection rises with host age to reach a plateau. For most (but not all) GI nematodes, intensity of infection, however, shows a different pattern with levels to infection rising but then decreasing from late childhood/teenage years with progression to adulthood 1,2. In addition within infected populations, there is further diversity in that infection levels vary between individuals and this does not follow a normal distribution but an over dispersed or skewed pattern. Thus, most individuals will harbor low levels of parasites, but a few will have high worm burdens. Furthermore, studies in humans following reinfection after drug clearance of worms have indicated predisposition to infection in which individuals appear to re-acquire infection levels similar to those that they originally had 1. Numerous studies have strongly supported a role for acquired immunity in humans to STH for many if not all species in generating the patterns of infection observed 1,3. However, the data also clearly indicate in simple terms that under natural infection (antigen challenge) protective immunity is not as effective as it might be and often only partial at best.

The concept of antigen challenge with regards to GI nematode infection is interesting to consider. For most species, the strategy evolved by these nematodes to maximize transmission is to seed the external environment with as many infective stages as possible. Millions of nematode eggs are shed via female worms within the host intestine that pass into the external environment and develop into millions of infective ‘embryonated eggs’ or for some species, infective larvae (that are for the most part relatively short-lived). Although infection can have seasonal fluctuations exposure to infectious stages, therefore, especially in subtropical and tropical regions of the world tends to be year round. Taken together with other observations, the generally accepted view is that GI nematode infections are acquired via repeated, relatively low doses of infection over prolonged periods of time. Thus, the antigen challenge will be varied in terms of the antigens involved, in terms of complexity and quantity, and derived from both established parasites and newly invading stages. This will also be accompanied in many cases by mechanical tissue damage.

The very fact that for most GI nematode infections increasing host age (and continued exposure to infectious stages) does not directly translate to ever increasing parasite burdens supports the hypothesis that acquired resistance does arise in some form if not in terms of complete sterile immunity. It is certainly true that the adaptive immune system responds to infection as most readily evidenced by numerous studies demonstrating raised levels of parasite-specific class switched antibody levels and from more recent studies, peripheral blood antigen-specific lymphocyte recall responses and cytokine production 4. Signature immunological features associated with GI nematode infection include those most often associated with immune-mediated disease such as atopy and allergy, namely elevated peripheral IgE antibody levels and peripheral eosinophilia 5–7.

## Laboratory models of immunity to GI nematodes

The very fact that natural infection via repeated and varied levels of antigenic challenge is superimposed upon multiple other variables including co-infection, behavior, nutrition, and parasite/host genetic heterogeneity has, of necessity, driven investigation of mechanisms underlying immune responses to GI nematodes into the laboratory and the utilization of parasite species naturally found infecting rodents in the wild.

The main thrust has been to study GI nematode species that are easy to handle and maintain in the laboratory but also those that bear some similarity to species infecting man or economically important domestic stock. Those most notably studied are *Nippostrongylus brasiliensis*, a natural ‘hookworm’ of the rat but studied extensively in the mouse, *Heligmosomoides polygyrus bakeri*, a natural roundworm infection of rodents including mice, *Trichuris muris*, a natural whipworm infection of rodents including mice, and *Trichinella spiralis*, a parasite with a cosmopolitan host distribution (including rodents) and atypical life cycle for GI dwelling nematodes but with a well-defined intestinal dwelling phase.

While these species have a common feature in that they have an intestinal phase of infection that under natural conditions is likely to be long lived (except for *T. spiralis*), they arrive in the intestine via different routes, at varied life cycle stages and occupy different intestinal niches. Although it is now abundantly clear from many studies that there are strikingly common features to all the infections in terms of host response (reflecting the signature immune responses seen in natural animal and human infections), there are also differences, which have arisen through variation in intestinal niche and life cycle, which should be borne in mind. For example, the natural mode of infection of *N. brasiliensis* is via skin penetrating L3 larvae from the external environment and then passage through the vasculature, penetration across the airways and subsequently movement into the GI tract via swallowing, into the lumen of the small intestine. Here, the parasites mature into adults, mate and females release eggs that pass out with the feces. *H. polygyrus bakeri* infection proceeds following ingestion of free-living L3 larvae from the environment that then penetrate the submucosa of the small intestine, molt, and then re-emerge into the intestinal lumen of the small bowel remaining coiled around the villi. Following mating, eggs are again shed from the intestine via the feces. Both these species are used as models of human hookworm infection based upon similarity of infection mode e.g. skin penetration or site within the GI tract although differences between model and natural infection clearly exist. For example, neither causes the punctate hemorrhages to the intestinal mucosa or associated anemia seen in human hookworm infection. *T. spiralis* is highly unusual as infection is initiated through ingestion of L1 larvae found within the muscle of a previously infected host. The larvae invade epithelial cells of the small intestine rapidly pass through the series of molts to become adult parasites by approximately 30 h. This parasite then produces live L1 larvae, which do not pass out of the host, but move via the lymphatics and blood to striated muscle where they invade myocytes and modify their biology to become ‘cysts’ in which the L1 live and grow until subsequent ingestion via the next host. The value of the *T. spiralis* model for study of mucosal immunity lies in its ability to stimulate a robust intestinal response activating many components of protective intestinal immunity.

A group of nematodes related to *T. spiralis* are the whipworms. Human whipworm (*Trichuris trichiura*) is one of the major species of STH of man and unusually, for species of GI nematodes studied in the laboratory, the rodent infecting species (*T. muris*) is extremely closely related phylogenetically and genetically 8. There are in fact many species of *Trichuris* that infect a remarkably wide variety of vertebrate hosts 9. All of them share a similar life cycle that begins upon ingestion of embryonated eggs from the external environment. Upon hatching within the GI tract, the L1 larvae invade the intestinal epithelium with a preference for the cecum and proximal colon. Here, they remain embedded within the epithelial layer progressing through molts until sexual maturity when their posterior ends protrude into the gut lumen to facilitate mating and egg deposition by the female parasites. The time this takes depends upon species. Other members of this family of nematodes that share many of these life cycle features within mucosa are the capillarid or pseudocapillarid nematodes of birds, reptiles, and fish thus confirming the Trichuroid nematodes as a very successful group of parasitic helminths of animals including man.

## The *Trichuris* system: as a paradigm of GI nematode infection

**Figure 1:**
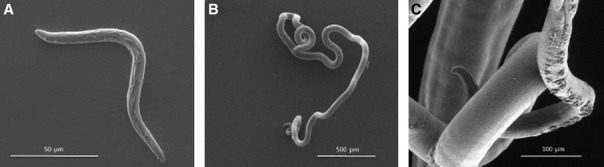
Scanning electron micrographs of Trichuris muris. (A). L1 larvae (days 0–9/11 postinfection), which are found embedded within epithelial cells of the cecum or colon, initially toward the base of the crypts of Lieberkühn. Note lack of slender ‘whip-like’ anterior end. (B). L3 larvae (days 21- 24-28 postinfection), again lacking a defined slender whip-like anterior morphology. (C). Adult (days 29–32 postinfection onwards). Note slender anterior end (with cuticular cephalic glands of unknown function), which would be embedded within the epithelial cells at the crypt table in the cecum or colon. The large posterior end would extend free into the intestinal lumen to facilitate mating and egg deposition by the female worms. Images taken by U. Rössler and T. Starborg, Faculty of Life Sciences, University of Manchester.

The use of the *T. muris* system (*Fig. *1) experimentally has increased over the last 15 years and has been informative in the identification and characterization of a number of aspects of immunity to GI nematodes and the wider field of fundamental immunology. This is not only because it is a tractable model that is easy to use within the laboratory. As stated above, it is one of the few rodent species of GI nematode with a clear human counterpart in terms of biology and exhibits a spectrum of immune responses and resistance that can be manipulated by host genotype (mouse strain), infection dose, and immunological intervention. Studies of a variety of inbred strains of mouse showed a spectrum of parasite expulsion, with most expelling parasites given as a moderate or large bolus before patency (parasite sexual maturity and successful egg production) was achieved, i.e. as larval stages of the nematode, although the speed of worm clearance varied between strains. However, a few strains did not expel their parasites and retained a full parasite burden. Moreover, early work investigating differences in resistance and susceptibility to *T. muris* between mouse strains showed a strong functional association between activation of T-helper 2 (Th2) cells and Th1 cells, respectively (reviewed in 10–12). These studies in our lab and others have paved the way for greater exploration and definition of both induction of immunity and regulation of immunity during chronic GI infection. The system also provided a distinct but comparable system to that of the protozoan parasite *Leishmania major*, which was groundbreaking in defining our fundamental understanding of cytokine-mediated immunity 13. Finally, infection of mouse strains with a low dose *T. muris* infection (tens of eggs or less) will result in a chronic infection. This is associated with the development of a Th1 response as seen on those few susceptible strains following high dose infection. The low dose reflects, at least in part, the naturally susceptible phenotype seen in the wild and experimentally repeated low dose (trickle) infections of *T. muris* will slowly generate resistance over time although not always complete resistance 14,15. Thus, this system has particular characteristics that make it a particularly powerful laboratory system to study. This ‘concomitant’ type of immunity has been discussed in relation to helminth immunity in general over many years 1.

The present review uses the *T. muris* system as a focus for discussion of our current knowledge of immune responses to GI nematodes highlighting areas of ignorance, controversy, and debate together with some suggestions for interesting areas for future investigation. We also aim to concentrate on the most pressing and recent areas of investigation. Where relevant, comparisons are drawn from other GI nematode studies and systems and where these systems are at the forefront of particular areas of our understanding.

## Innate responses to GI nematodes

The distinction between innate and adaptive immune responses is becoming blurred as important new ‘innate’ cell populations are defined and responses to antigen challenge are considered at the level of barrier cells such as epithelial cells. With regards to mucosal surfaces and GI dwelling nematodes, this has been a particularly active area of research whereby the interplay between intestinal epithelial cells, new populations of innate lymphoid cells (ILCs), myeloid antigen-presenting cells (such as dendritic cells and intestinal macrophages) is generating new insight into how early events can both lead to control of the infection and influence the subsequent adaptive arm of immunity.

### The first line of intestinal defense: the secreted mucus barrier

One aspect of the intestinal mucosal barrier that often gets overlooked is that of the secreted mucus layer. This layer is the first barrier component GI nematodes will come into contact with during the intestinal phase of infection, either as a larval stage during the early infective process or as adult stages during the reproductive phase of the infection. The intestinal niche occupied by the particular species of worm will dictate the type of mucus barrier encountered, but the barrier is dynamic and varies throughout the GI tract being up to 70 μm in depth overlying parts of the large bowel epithelium 16. Thus, GI nematodes will interact with the mucus layer and in many cases have to traverse through it to get to the epithelial layer or thrive and reproduce within it. At all sites, the mucus layer presents as a highly ordered hydrated gel formed of mucins, large heavily glycosylated glycoproteins of high molecular weight, secreted from goblet cells (GCs), that interact with anti-microbial compounds, antibodies, commensal metabolites, etc. Different mucosal sites around the body vary in terms of the dominant secreted mucin being produced. In the intestine of both mouse and human, Muc2 dominates 17.

The mucus layer has been considered as primarily part of the innate immune response, but data have shown that mucin production is regulated by many components of the immune system including the adaptive arm. Of particular note are the type 2 cytokines interleukin-4 (IL-4) and IL-13, the latter playing roles in proliferation and differentiation of GCs 18,19.

Early during a primary intestinal infection, it is self-evident that the mucus barrier does not prevent establishment of infection. However, as infection proceeds, a GC hyperplasia has been observed in many systems in which resistance and worm expulsion from the gut followed including *N. brasiliensis*, *T. spiralis,* and *H. polygyrus bakeri* 20–22. This was shown to be under T-cell control 23 and subsequently confirmed and extended to show an influence of IL-13, presumably CD4^+^ T cell derived (i.e. Th2) in controlling this process 19,24. Following, *T. muris* infection resistance was associated with an increase in Muc 2 containing GCs and secretion of elevated levels of Muc 2 under IL-13 control 25. Moreover, infection of Muc2-deficient mice led to a delay in worm expulsion suggesting an important if not essential role for this mucin as a component of the secreted mucosal barrier 25. Surprisingly, even in the absence of Muc2, some GCs were observed to secrete mucins, which were identified as Muc5ac, a mucin not normally found in the intestine 25,26. This was under the control of IL-13 production and in Muc5Ac's absence, mice were unable to expel a primary *T. muris* infection. Also, loss of the Core 2 β1,6-N-acetylglucosamineyltransferase (C2GnT) enzyme C2GNT1 impairs worm expulsion following a high dose infection, possibly through modification of mucin glycosylation 27. It is noteworthy that acute *T. muris* infection is also associated with intestinal upregulation of GABA-α3 receptor, a gene associated with hyper secretion of mucus, Math-1, the enterocyte transcription factor for differentiation toward absorptive cell lineage and Spedef, the transcription factor involved in terminal differentiation of GC. Acute infection was associated with strong expression of D-GalNac, a-2, 3-sialic acid, and highly charged mucins. The carbohydrate-rich glycocalyx that is found at the surface of the epithelial cells also undergoes change with increases in cell surface mucins Muc4 and Muc13 increased in acute infection 28.

Investigations further demonstrated that Muc5Ac was elevated in the intestine only in infected mouse strains where resistance to *T. muris* was apparent and not in SCID mice 26, the latter suggesting that if ILC2 responses are initiated in SCIDs following infection, they do not induce MUC5ac expression. Interestingly, *T. muris* itself was shown to secrete proteases that were able to degrade Muc 2 but not Muc5ac 29. As a counter effect, the infected intestine in this system was also shown to upregulate a number of serpins suggesting a co-ordinated and complex response at the host/parasite interface 29. Recent work has added to this area and shown that IL-22, a cytokine often associated in epithelial repair, contributes to the intestinal GC response following both *N. brasiliensis* and *T. muris* infection. IL-22 null mice showed altered mucin expression and delayed worm expulsion in both systems. Surprisingly, however, data from *in vitro* studies did not show any role for IL-22 in enhancing Muc5Ac expression 30. It is also noteworthy that GCs also secrete a number of other factors associated with resistance including the resitin-like molecule RELMβ 31 and indolamine 32, which can increase the turnover of intestinal epithelial cells that contributes to removal of the worms from the gut, at least in the case of *T. muris* 33.

The importance of RELMβ in protection against GI nematodes appears to be varied. Studies suggest that where the parasite lives within the lumen of the intestine, RELMβ can play a protective role, possibly by interfering with parasite feeding or development 34. However, epithelial dwelling parasites such as *T. spiralis* and *T. muris* do not seem to be critically affected by RELMβ as RELMβ null mice are able to expel parasites effectively 34,35. However, similar to the case of *T. muris*, Mu5Ac null mice do show deficiencies in their ability to efficiently expel both *T. spiralis* and *N. brasiliensis*. The mechanism whereby Muc5Ac mediates protection is unknown, but may operate directly on the parasites 26.

With regards to *T. spiralis* infection, expulsion of a primary infection is associated with a GC hyperplasia which may contribute to protective immunity as described above 36. However, a pronounced intestinal mast cell hyperplasia is also apparent and there is substantial data supporting a protective role for these cells via the secretion of specific proteases that contribute to intestinal inflammation including changes in epithelial integrity 37–40. These observations highlight the differences in niche occupied by the parasites and thus may influence whether particular immune effector mechanisms operating at mucosal surfaces are more or less effective.

The intestinal GC response during secondary and subsequent infections (e.g. trickle infections) has received less attention. Early studies of multiple *N. brasiliensis* infections in rats suggested significant changes in these responses following challenge in both the intestines and the lungs 41–43 remembering that infection is via skin penetration and migration through the lungs. Indeed, recent work suggests that immunity to secondary infections for GI nematodes utilizing this infection route primarily operates at pre-intestinal phases (larval stages) including both skin and lungs 44–47.

Resistance to challenge with *H. polygyrus bakeri*, however, has been the most often studied system in this context as a primary infection in most strains of mouse is long lived and protective immunity does not readily develop. This does not quite represent the natural challenge as adaptive immunity is induced using an abbreviated infection (following use of an anthelmintic or via primary infection with irradiated L3 larvae that do not mature to adult parasites), but does prime for efficient protection 48–51. In this system, GC hyperplasia is observed with GC-derived RELMβ playing a protective role 34, although the contribution of secreted mucins or the mucus gel to resistance has not yet been defined. The fact that *H. polygyrus bakeri* spends part of its early intestinal occupation embedded in/below the muscularis externa provides the host with a potential new niche to encounter and respond to the parasite 52. Indeed, during a challenge infection, larval stages are controlled, at least partially by trapping within type 2 granulomas at this site 53.

In the *N. brasiliensis* and *T. spiralis* system in rats, following priming with large bolus of infective stages, a secondary infection of a similar parasite load was expelled rapidly and in some cases within 24 or 48 h. Observations suggested that intestinal mucus could play a role, perhaps by ‘trapping’ parasites 54,55, although the nature of the mucins involved is not known.

The *T. muris* system is one of the few where changes in the mucus barrier have been investigated during chronic infection. In contrast to acute infection, no upregulation of Math-1 or Spedef is found, but the transcription factor Hes-1 is upregulated, reflecting the increase in crypt size and cell number during prolonged infection. No increase in cell surface mucins Muc4 and Muc13 is seen during chronic infection, but an increase in Muc17 is found during prolonged infection. A marked change in glycosylation of secreted mucins was also observed following chronic infection, with low expression of D-GalNac when compared to acute infection. Also, the mucins during chronic infection were sialylated rather than sulphated and much less charged than those seen during acute infection 28. Changes in the mucus barrier, particularly during chronic *T. muris* infection could have major influences upon the microflora populations of the cecum affecting both the microbial populations found and their access to intestinal epithelia. Immunoregulatory consequences that follow on from this have yet to be investigated, but are clearly important to define. Other studies have already identified changes in microflora during prolonged intestinal helminth infection including following *Trichuris* infection 56.

## Innate lymphoid cell responses to GI nematodes

The definition of CD4^+^ T cells as major producers of cytokines 57 confirmed their role as key regulators of many innate effector mechanisms operating during GI nematode infection. Alongside, technological advances in flow cytometry re-evaluation of the cytokine-mediated regulation of immune responses have taken place especially in the gut and associated lymphoid tissues following GI nematode infection.

There have been a number of studies showing varied effects of different innate cell types following GI nematode infections such as NK cells 58 and γδ T cells 59,60. The discovery of new lymphoid cell types (ILCs) has been particularly enlightening in this respect 61. ILCs have been placed into at least three functional groups 62. The subset that dominates following GI nematode infection belongs to the ILC2 group. The notable feature of these cells is their capacity to produce substantial levels of type 2 cytokines including IL-13, IL-5, and IL-9. These cells have been identified in the lineage negative fraction of lymphoid cells both within intestinal tissue, draining secondary lymphoid tissue, and adipose tissue 63–65.

The source of IL-13 that regulates the GC response is likely to be the CD4^+^ T cell, the ILC2, or both. Whether the different cell types contribute to different phases of the response are unknown. It may well be that ILC2-derived IL-13 initiates the response and CD4^+^ T cells contribute once the response is underway, although this is far from clear in any GI nematode system at present. The potential for the ILC2 to contribute is significant, as on a cell-for-cell basis, they are believed to secrete considerable amounts of cytokine 66 and in a non-antigen-specific manner.

As mentioned above, most of the GI nematode systems studied have used models whereby large bolus infections are given to induce resistance (type 2 immunity) and worm expulsion. In this situation, ILC2s have been shown to be present in increased numbers both in the gut tissue and in the draining lymphoid tissue (e.g. MLN) 67–69. Furthermore, increasing the numbers of ILC2s via cytokine injection *in vivo* or transfer of *in vitro* generated ILC2s demonstrated convincingly that IL-13 derived from ILC2s is capable of mediating worm expulsion via whatever effector mechanism is required for the particular nematode in question 67,69. This is distinct however from showing that ILC2s play a role in mediating immunity to GI nematodes following ‘natural’ infection. The clearest evidence for this has come from work by Andrew McKenzie's group and the *N. brasiliensis* system in which *Rora*^sg/sg^ mice show impaired expulsion of parasites from the gut. *Rora* is the transcription factor critical for the development of ILC2s. The capacity of the ILC2s to produce IL-13 and generate a GC response following high dose challenge was evident in WT mice, but diminished in the *Rora*^sg/sg^ mice despite having normal levels of CD4^+^ T cells in the draining MLN 70. These findings are partly mirrored by recent work investigating the role of the transcription factor Gif1, whereby, deletion of this gene renders mice susceptible to a high-dose infection with *N. brasiliensis*, with deficiencies seen particularly in lung-associated ILC2s. Interestingly, in gut associated lymphoid tissue, ILC2 cells were still able to increase in number following *N. brasiliensis* infection of Gif1 null mice and there was an associated depression in CD4^+^ T cells in the draining lymph node 71.

Data from the *N. brasiliensis* system furthermore suggest that numerically ILC2 cells comprise the major source of IL-13 following infection, out-numbering CD4^+^ T cells 67. Whether this holds true for all systems as yet remains unclear, and there appears to be some degree of variation. For example, IL-13^+^ ILC2 levels in the MLN following *T. spiralis* infection reflect those seen following *N. brasiliensis* infection although do not necessarily outnumber IL-13^+^ CD4^+^ T cells (Lawrence and Grencis, unpublished data). In terms of *H. polygyrus bakeri* infection, a modest rise in ILC2 has been shown in both MLN and spleens early (day 6) following a single infection 72. This is a time when larval parasites are embedded in the gut submucosa and have not returned to the intestinal lumen to begin the protracted phase of infection. At a slightly later time point (day 10), an elevation in numbers of ILC2s is seen in the MLN and is higher in mouse strains that do go on to expel a primary infection 73. In the case of the drug-abbreviated resistance model of *H. polygyrus bakeri*, the host protective role of ILC2s remains to be determined. Following high dose *T. muris* infection, the magnitude of the ILC2 response appears much less pronounced than seen in either *N. brasiliensis* or *T. spiralis* infections. Moreover, following a high dose *T. muris* infection, it would appear that the major IL-13 producing population in the MLN is the CD4^+^ T cell (Lawrence and Grencis, unpublished data). Again, however, transfer of *in vitro* generated ILC2 populations that produce IL-13 accelerates the expulsion of *T. muris* from the gut 69. Intriguingly, recent studies have shown that RAG^−/−^ mice deficient in vitamin A expand the numbers of IL-13-producing ILC2 following even low-dose infections of *T. muris* and that this is reflected by increased GC numbers. The elevated ILC2 numbers are associated with accelerated worm expulsion from the intestine strongly supporting a role for ILC2-derived IL-13 in promoting worm removal. Overall, these studies suggest that ILC2s can act as sensors that can respond to nutritional changes and respond accordingly enhancing gut barrier defenses when CD4^+^ T-cells responses are less than optimal 74.

Whether the differences observed between different GI nematode systems studies reflect technical differences used in defining these relatively small populations of cells, the presence of subsets within the ILC2 population, differences in experimental design, or truly indicate that the host fine tunes the Type 2 protective response depending on the precise nature of the challenge in the gut remains to be defined. It is clear that ILC2s form a significant component of the innate response in the mucosa in response to parasite challenge. The data so far would suggest that this is observed most robustly following a large dose of infection and in such a case may contribute significantly to worm removal via IL-13-controlled responses. Available data from *T. muris* low dose infection studies or primary *H. polygyrus bakeri* studies would indicate that whatever levels of ILC2 are generated, they are not capable of mediating parasite expulsion, from a primary infection. Clearly, many aspects of regulation of chronic GI nematode infection remain to be defined. It may well be that the parasites have evolved mechanisms to downregulate the early expansion of ILC2s. In this regard, recent studies from the *H. polygyrus bakeri* system have shown a role for IL-1β in downregulating early ILC2 responses. Absence or depression of IL-1β levels leads to elevation of type 2 protective responses and worm expulsion 72. This is not the case in *T. muris* infection, where IL-1β null mice were susceptible to a high-dose infection unlike WT mice that expelled their worms. Infected IL-1β null mice were unable to polarize CD4^+^ Th2 cells, although ILC2s were not assessed 75.

The generation of fully functional ILC2s appears to be dependent upon precursors receiving a set of signals from epithelium and perhaps other cell types. The cytokines, IL-25, IL-33, and thymic stromal lymphopoietin (TSLP) assume key roles here and in mice, precursors of lineage negative (ILC2) that can respond to IL-25/33 and helminth infection are found in many tissues around the body 61,68. Although all three cytokines are thought to be epithelial derived, other signals from gut epithelium are also believed to be important. Epithelial Act1 (an NFκB activating protein) is key to IL-25-mediated expansion of ILC2s 76 as is Trefoil factor 2 for induction of IL-33 in lung epithelia 77. Many studies have now confirmed that both IL-25 and/or IL-33 can expand populations of ILC2s both *in vitro* and *in vivo* 68,69,78. Moreover, such cell populations are also present in a variety of immunodeficient mouse models although their function can be compromised without signals generated from immune cells 67,68.

### IL-33

IL-33 has received particular attention as an alarmin and can be released from damaged epithelial cells 79 and is, therefore, well placed to initiate rapid changes following GI nematode infection. IL-33 mRNA expression is also upregulated in the intestine following intestinal GI nematode infection 78,80 and exogenous administration of IL-33 promotes many IL-13-mediated effector mechanisms including GC hyperplasia and RELMβ production that ultimately leads to worm expulsion 78. Moreover, IL-33 null mice have impaired ability to expel some GI nematode parasites 81,82. Also, recent work suggests that *H. polygyrus bakeri* releases products that suppress ILC2 responses via downregulating IL-33, suggesting modulation of IL-33 is an important component of the protective response 72.

**Figure 2:**
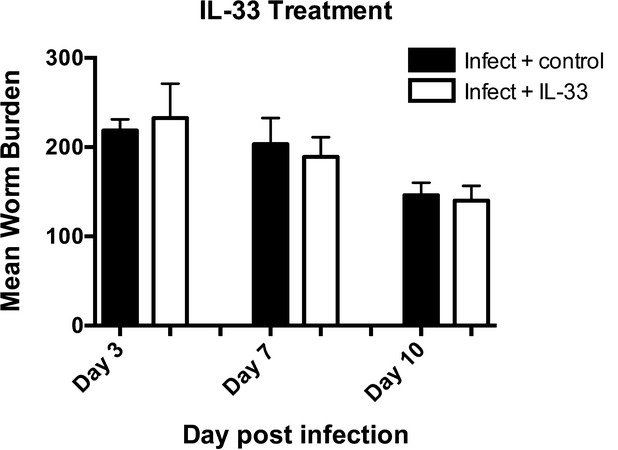
Interleukin-33 (IL-33) does not accelerate intestinal expulsion of *Trichinella spiralis*. Exogenous IL-33 (0.8 μg) or PBS 78 was injected intraperitoneally into C57BL/6 male mice on days −1, 2, 4, and 6 postinfection. Intestinal worm burdens were measured on days 3, 7, and 10 postinfection. Values represent mean ± SE for 2–5 animals per group.

Current data would suggest, however, that a critical role for IL-33 may not apply to all GI nematode infections. For example, ST2 (IL-33R)-deficient mice are still able to expel *N. brasiliensis* 83, although IL-33 null mice show an impaired expulsion of this parasite 81. ST2 null mice can expel *T. spiralis* 84 and *T. muris* (authors' unpublished data) effectively and IL-1RAcP null mice (part of the IL-33 R) are also able to expel *T. muris* effectively 75. Following both *T. muris* and *T. spiralis* infection, the absence of MyD88 (through which IL-33 signals via ST2) does not affect worm expulsion and animals make effective type 2 cytokine responses 84,85. Nevertheless, exogenous IL-33 has been shown to accelerate clearance of *T. muris* from the intestine from mice that would normally progress to chronic infection. Such treatment is effective when given early during infection, but is not effective when given once a chronic infection has established. Moreover, early IL-33 treatment of infected SCID mice displayed altered pathology, but did not induce worm expulsion mice suggesting that additional signals from the adaptive arm of the response are required for driving mechanisms underlying protection 78. Interestingly, similar *in vivo* IL-33 administration to *T. spiralis*-infected mice did not accelerate worm expulsion (*Fig. *2). Whether the responses required for protective immunity to *T. spiralis* do not require IL-33 or that the Type 2 response is already optimal remains to be defined. Certainly, *T. spiralis* has a life cycle strategy that is very different from almost all other GI nematodes and efficient parasite expulsion from the intestine may, paradoxically, be of real value to this species preventing potentially lethal levels of muscle larvae establishing in the muscles.

Thus, taken together, it would appear that although IL-33 is a potent inducer of ILC2s and can promote worm expulsion, it is not always necessary for generating protection. If it is involved, the available data suggest that it does not operate via ST2 and may require components of the adaptive response for optimum efficiency. Moreover, the capacity for IL-33 to operate as a direct nuclear transcription factor 79 may warrant further investigation.

### IL-25

In some senses, IL-25 mirrors many of the effects of IL-33 in studies of immunity to GI nematode infection. IL-25 mRNA is upregulated in the intestine following infection of *N. brasiliensis*, *H. polygyrus bakeri*, and *T. muris* 68,69,80,86. IL-25 null mice show a delayed expulsion of *N. brasiliensis* 68,87 and resistance is abrogated in mice resistant to *T. muris* 86. Both systems show a decreased type 2 cytokine response accompanying susceptibility. With regards to *T. muris*, *in vivo* administration of IL-25 enhances type 2 immune responses and promotes resistance in susceptible strains but not in SCID mice 86. This is similar to that seen after IL-33 treatment again suggesting worm expulsion is dependent upon components of adaptive immunity. Also, in the absence of an adaptive response, treated and infected SCID mice develop enhanced inflammation following either IL-25 86 or IL-33 78 treatment. In contrast, IL-25 can also play an anti-inflammatory role in addition to enhancing type 2 responses 86.

### TSLP

TSLP is believed to play a major determining role in type 2 immunity. This factor is produced by a variety of cells types, particularly epithelial cells, keratinocytes, and stromal cells but also mast cells and DCs. TSLP is also known to have a variety of functions although in particular it has been suggested to license DCs to induce Th2 responses 88. In terms of helminth infection, TSLP has been shown to play a major role in protective immunity to *T. muris*. TLSP is induced in the intestine upon infection and TSLPR null mice are unable to expel their intestinal parasites compared to WT and have diminished type 2 cytokine responses. Furthermore, it was shown that TSLP inhibits the production of IL-12/p40 by dendritic cells thus attenuating the induction of Th1 responses and IFN-γ production important for progression to chronic *T. muris* infection. Importantly, however, neutralization of IFN-γ in susceptible TSLPR null mice infected with *T. muris* allowed the development of type 2 responses and worm expulsion suggesting that TSLP was not an absolute requirement for induction of type 2 immunity 89. Interestingly, TSLP activity is also not necessary to drive type 2 responses following *N. brasiliensis* and *H. polygyrus bakeri* infections as shown by studies in TSLPR null mice. Indeed, these parasites secrete products that are able directly inhibit the production of IL-12/p40 by dendritic cells 90. Thus, TSLP may rather play a prominent role in the attenuation of Th1 and Th17 responses 90 and indirectly license type 2 cytokine responses. The reasons for the differences between different parasites are unknown, but it may well be that *T. muris* (unlike the other two species) has evolved a strategy whereby induction of a Th1 response is important to the success of chronic infection and inhibiting the ability of DCs to do this would be disadvantageous for the parasite.

It is tempting to suggest that following natural infection by helminths (i.e. low numbers), the levels of key cytokines such as IL-25, IL-33, and TSLP do not individually or collectively reach sufficient thresholds to enable large enough numbers of ILC2s to generate the effector mechanisms that can remove the parasites. The inability to do this may be part of the parasites' immune evasion strategy. Thus, during initial encounter, this aspect of the innate response is simply not strong enough to eradicate the parasites. Experimentally, if you enhance the ILC2 response in any way, parasites are controlled, primarily through provision of large amounts of IL-13, a cytokine central to many of the anti-GI nematode host protective effector mechanisms.

As infection progresses, presumably the ILC2 response increases, but this occurs alongside initiation of the adaptive arm of the response with production of antigen-specific CD4^+^ T cells. There is abundant evidence from many studies indicating that CD4^+^ T cells play a major role in mediating worm expulsion 91–94. Moreover, movement of CD4^+^ T cells to the intestine is critical for CD4^+^ T-cell-mediated control of infection as shown by studies in *T. muris* 95. The advantage that CD4^+^ T cells have (in addition to generating memory) is that they are antigen specific and will only be activated in response to antigen, potentially limiting pathology 96,97. Also, as they are antigen driven, they can clonally expand, move to the site of infection, and continue to function until the antigen is removed. If the capacity to expand ILC2s is limited then the clonal expansion of the CD4^+^ T cells may then come into its own. The construction of mouse models where it is possible to temporally and specifically remove ILC2s *in vivo* in conjunction with mice where CD4^+^ T cells can similarly be removed will undoubtedly confirm the importance of ILC2s during primary infection with GI nematodes and identify the contribution of the adaptive (CD4^+^ T cell) response. Recent data have indicated that ILC2s may present antigen to CD4^+^ T cells in a class-II MHC-restricted manner 98 and support the view that the capacity of ILC2s to mediate worm expulsion relies on the presence of CD4^+^ T cells to maintain optimal ILC2 function. Also, recent work suggests that ILC2s serve to enhance the CD4^+^ Th2 cell response 99.

A complete unknown is the functional importance of ILC2s following resistance that is slowly built up following trickle infections. It may well be that under repeated challenge that production of IL-13 from ILC2s does make a significant contribution to the removal of incoming parasites. However, in this scenario, the ongoing CD4^+^ T-cell response would also produce IL-13 and data from the *N. brasiliensis* system suggest that the CD4^+^ T cell is critical to control of secondary infections 100. Perhaps, the critical factor is the number and size of infection events. The nature of these will dictate the relative contributions of each of the IL-13-producing cell populations. Overtime, the net effect would be the same: type 2-mediated protective immunity will establish itself, though not completely, because some of the parasites will have effectively evaded immunity and established patent infection.

ILC2 responses may be particularly effective at promoting protective anti-GI nematode effector responses where a rapid response is required in the mucosa without the need for antigen specificity, such as muscle contraction, epithelial turnover, or production of particular mucins and anti-microbials such as RELMβ from GCs. In the gut, the epithelium is a particularly attractive target tissue as it constantly undergoes renewal replacing itself every few days. Differentiation of both the secretory and the non-secretory epithelial cell types is controlled within this time frame. Interestingly, in relation to the gut, in addition to large amounts of IL-13 (and IL-5), amphiregulin has recently been highlighted as a secreted product of ILC2s 101. Amphiregulin was identified previously as an important component of the protective response to *T. muris*, particularly in relation to accelerated epithelial turnover 102,103 and suggested to be derived from, at least in part non-T-cell innate sources 102.

Overall, the innate intestinal response to gut nematode infection still remains to be comprehensively described. As additional important cell populations in the hemopoietic system are precisely defined, we will be well placed to delineate their different roles and their interplay in the host response. For example, the recent identification of an extramedullary population of hematopoietic progenitor cells that is driven by TSLP during Type 2 allergic type responses (including following *T. muris* infection) suggests that this population particularly comprises granulocyte monocyte progenitors. Upon adoptive transfer of expanded TSLP-elicited cells of this type, it was shown that they influenced many aspects of the anti-*Trichuris* response including GC hyperplasia, elevated serum IgE, and accelerated worm expulsion from the gut 104.

## Induction of the adaptive response to GI nematodes

The recently demonstrated ability of ILC2s to present antigens to CD4^+^ T cells and enhance Th2 responses suggests a role for induction of adaptive responses. However, the nature of GI nematode antigen encounter, processing, and presentation remains a contentious area *per se*. The intestinal DC is extremely well placed and equipped to undertake this function. Definition of intestinal DCs has undergone a re-evaluation over the last few years as it was recognized that similar levels of CD11c and MHCII expression could be seen on both bona fide DCs and intestinal macrophages. Broadly speaking, the consensus is that in the mouse gut (largely from studies of the small intestine) that there are three major DC subpopulations present in the *lamina propria*. CD103^+^ CD11b^+^ and CD103^+^ CD11b^−^ DCs that are derived from the same committed DC precursor and a third CD103^−^ CD11b^+^ population whose origin is unknown 105.

Many of the studies on the role of DCs during GI nematode infection were carried out before the most recent consensus definitions, but have shown that the DC is critical to the generation of immunity to gut nematodes. Intestinal epithelial cell-intrinsic IKβ kinase (IKK)-B-dependent gene expression is a critical regulator of responses of DCs and CD4^+^ T cells during *T. muris* infection in the gut. Zaph *et al*. 106 showed that intestinal epithelial cell activation of the NFκB pathway influenced the ability of intestinal DCs to generate a Th2 response. This effect was dependent upon conditioning by epithelial-derived TSLP that served to modulate pro-inflammatory cytokine production by DCs and others. The role of TSLP during infection was particularly important in limiting IL-12p40 production by DCs 106. TSLP conditioning was shown not to be important for infections with other GI nematode species such as *N. brasiliensis* and *H. polygyrus bakeri*, whereby the parasites themselves interfere with IL-12p40 production by DCs directly 90. This may be related to either differences in site of infection (small versus large intestine) or the fact that the strategy to progress to long-term infection is different between the species. Nevertheless, in *T. muris* infection, movement of DCs is implicit in their ability to initiate host protection. Intestinal CD103^+^ DCs need to migrate to the epithelium via epithelial derived chemokines to acquire antigen, prior to priming T cells in the draining MLN. Indeed, the speed of migration to the epithelium appears to influence the development of subsequent response, i.e. resistance or susceptibility 107. A role for the pattern recognition receptor (PRR) Nod2 also plays a role in this process suggesting that either sensing parasite antigens (remembering that *Trichuris* burrows into epithelial cells) or commensal products that will have gained access to the epithelium influence the subsequent response to the worm 108. The recent demonstration that in the small intestine, soluble antigens from the lumen are taken up by CXCR1^+^ macrophages and transferred to CD103^+^ DCs to induce tolerance and T-regulatory cell differentiation 109 begs the question of whether soluble GI nematode antigens follow this route too? GI nematodes secrete a plethora of soluble antigens some of which are produced in the gut lumen and presumably access such pathways.

The importance of intestinal DCs in regulating the adaptive response to GI nematodes is well established and changes in CD11c^+^ DC populations are known to occur during both acute and chronic infecting species. Following *N. brasiliensis* infection, DCs numbers expand in the MLN, although there is a decrease in the CD86^hi^ CD8α^int^ CD11b^−^ subset coincident with lower levels of CD40, CD86, and CD103 expression 110. Recent work has identified a population of PDL2^+^ CD301^+^ DCs in the MLN (and indeed the mediastianal lymph nodes draining the lungs) following infection with *N. brasiliensis* that are important for the induction of Th2 responses. These DCs were dependent upon expression of the transcription factor IRF4, which is involved in a number of Th2-related responses 111.

Following long-term infection with *H. polygyrus bakeri*, CD8α^int^ DCs numbers fall to very low levels accompanied by changes in capacity to secrete a number of cytokines 110. Furthermore, a population of CD11c^lo^ DCs is found that, at least *in vitro*, did not optimally stimulate antigen-specific CD4^+^ Tcells to proliferate and produce effector cytokine, but did induce higher levels of FoxP3^+^ T cells in the presence of TGFβ 112.

Studies from the *T. muris* system have identified an important early role for DCs and TGF-β in establishing resistance or susceptibility to infection. Mice given a low dose of *T. muris* and treated *in vivo* with anti-TGFβ were significantly protected from chronic infection. Subsequent experiments showed that CD11c^+^ intestinal DCs that lack the TGF-β-activating integrin αvβ8 make an early and pronounced type 2 immune response to *T. muris* and expel parasites in an accelerated manner. This effect was dependent upon CD4^+^ T cells but not FoxP3^+^ Tregs 113. Collectively, these studies indicate that the intestinal DC compartment is profoundly modified upon GI nematode infection, but can dramatically influence the developing or ongoing immune response although many aspects remain to be defined.

Other work, however, has suggested that additional cell types play an important antigen-presenting role during GI nematode infections including the basophil and ILC2 98,99,114. Notwithstanding their possible role as antigen-presenting cells (APCs), granulocytes including basophils, eosinophils, and mast cells have also all been suggested or shown to have regulatory functions during GI nematode infection 115–120. Interestingly, GC have been identified as cells that can transfer antigen from the gut to other APCs 121, and more recently, intestinal DCs have been shown to acquire antigens together with mucins (Muc2) from the mucus layer overlying the epithelium 122. In both cases, the data suggest a role for these mechanisms in intestinal homeostasis and tolerance rather than T-cell activation. The possible roles of mucins and importance of these routes during GI nematode infection remain to be determined.

The recent re-examination of the macrophage lineage in the gut has highlighted important functions for these cell populations during intestinal GI nematode infection including protection and repair. A current working phenotypic definition of the *lamina propria* macrophage is a CD11c^+^, CD11b^+^, F4/80^+^, CD64^+^ CX3CR1^hi^ cell. Similar to intestinal CD103^+^ DCs, these cells appear to be continually renewed throughout adulthood from hematopoietic stem cells but via a Ly6C^+^ monocyte cell unlike intestinal CD103^+^ DCs 123. Interestingly, recent work has highlighted the capacity of macrophages to proliferate at tissue sites increasing numbers of cells over and above any recruitment. This is particularly evident during type 2 cytokine-dominated environments including following nematode infection 124. There appears to be different control of proliferation including IL-4Rα-dependent and -independent phases, although whether this reflects clear differential function remains unclear. *H. polygyrus bakeri* infection has been studied in this respect and provides an environment for such proliferation in the peritoneum, but whether this occurs in the intestine is unknown 125. This particular parasite invades the submucosa of the intestine and generates a strong tissue response in which alternatively activated macrophages (AAMs) are believed to play a protective role contributing to the neutrophil/macrophage-rich intestinal granuloma that forms around the developing larvae 126. Following *N. brasiliensis* infection, AAMs are believed to contribute the protective response via their activity on intestinal smooth muscle contraction 127. Following *T. muris* infections, changes in macrophage populations are seen in the intestine following both acute and chronic infection with greater numbers of cells accumulating during acute infection 128. A role for AAMs in resistance in this system remains to be proven, however, although if acting, it does not depend on arginase production, a signature factor associated with these cells 129. The broader functions of intestinal CX3CR1-high macrophages cells are growing and include roles in maintenance of intestinal FoxP3^+^ Tregs and regulation of intestinal homeostasis via crosstalk with intestinal epithelium. They also exhibit pro-inflammatory effects during intestinal inflammation although clear roles during GI nematode infection remain undefined.

## Chronic infection and host immunoregulation

While the contribution of GI nematode infection to the modified hygiene hypothesis has been the subject of much debate and investigation in both human and animal models, the mechanistic basis is still controversial. Given that infections are generally chronic in nature and that the majority of infected individuals do not suffer severe pathology, then regulatory mechanisms must be central to this debate both in terms of preventing worm expulsion and pathology. The majority of work has concentrated upon the role of regulatory T cells and to a lesser extent regulatory myeloid populations such as macrophages. The importance of the different cell populations and their roles in regulating host protection and pathology are still subject to debate.

### Regulatory T cells

There is supportive evidence for a role of ‘classical’ regulatory FoxP3^+^ T cells from a number of elegant studies of infected human populations 130–132. The work is of necessity correlative, but adds to experimental studies in a number of laboratory systems—most notably those that exhibit chronic infection i.e. *H. polygyrus bakeri* 133,134 and *T. muris* 113,135, where functional studies including the mechanisms of action via secretion of TGF-β and IL-10 have been explored.

Following chronic *H. polygyrus bakeri* infections, an increase in the number of FoxP3^+^ Tregs has been observed by a number of groups 134,136,137. However, their role in suppressing worm expulsion and pathology is less clear using different experimental approaches for removal of FoxP3^+^ Tregs via injection of antibodies to surface marker such as GITR or CD25 or the use of DEREG mice in which FoxP3^+^ Tregs are removed following injection of *Diptheria* toxin 134. There is also some evidence from the *T. muris* system using *in vivo* depletion of FoxP3^+^ Tregs with antibodies supporting a role in suppressing worm expulsion 135, although data following chronic infection in the DEREG mice suggest they do not play an essential role 113. Recent studies used the FoxP3DTR-GFP model 138 to delete FoxP3^+^ Tregs during the first 8 days or second 8 days following a low dose *T. muris* infection. Analysis of outcomes on day 35 postinfection supported a slightly greater role for FoxP3^+^ Tregs on moderating worm burdens if depletion was done over the first 8 days of infection. In this case, there were fewer worms in the depleted mice and evidence of raised Th2 responses and elevated total IgE levels. Nevertheless, all animals in both early and late depleted groups maintained low levels of infection suggesting that other cell populations contribute to the modulatory process or that in this depletion system, FoxP3^+^ Tregs recover sufficiently to downregulate the generation of protective immunity 138.

Data from both systems have added further complexity, however. The generation of FoxP3^+^ Tregs is dependent upon the production of active TGF-β. Fascinating data have shown that *H. polygyrus bakeri* secretes a TGF-β mimic that can induce the production of FoxP3^+^ Tregs *in vitro* 139. The precise mechanism whereby it gains access to differentiating CD4^+^ T cells given the parasite secretes the molecules in the intestinal lumen also remains to be determined. Given a number of recent publications showing how the mucosal barrier is much more dynamic than originally thought in terms of movement of molecules across the epithelium, it is not without precedent. Moreover, the work adds weight to a key role for the TGF-β pathway in chronic helminth infection 113.

Even though the data from *in vivo* depletion studies of FoxP3^+^ Tregs are not supportive of a critical role in the *T. muris* system, it has informed on an important role for TGF-β on induction of immunity. Intestinal CD103^+^ DCs in the large intestine express elevated levels of αvB8 integrin on their surface and conditional DC knockout mice for avβ8 have shown that this integrin is critical for production of FoxP3^+^ Tregs and in their absence severe pathology is apparent. A low level chronic infection of *T. muris* is rapidly expelled from these mice and accompanied by a major elevation of the Type 2 cytokine response 113,140. This is very supportive of a key role for TGF-β in regulating the early host protective immunity to the parasite although it does not marry with the observation that depletion of FoxP3^+^ T cells in DEREG mice during the early part of infection has little effect on subsequent worm burden (Klementowicz & Travis, unpublished observations). However, the interplay between DCs, T cells, intestinal macrophages, ILC2s, and epithelium remains to be comprehensively defined.

A GI nematode model that supports a key role for FoxP3^+^ Tregs is that of *Strongyloides ratti* in the mouse. In this system, removal of FoxP3^+^ Tregs (DEREG mice) was associated with enhanced type 2 immunity and accelerated worm expulsion. However, this effect was only seen when FoxP3^+^ Tregs were absent during the first few days of infection. In addition and perhaps surprisingly, no effect on gut pathology was associated with their depletion 141,142. A role for FoxP3^+^ Tregs cells in controlling pathology was shown following a primary infection with *H. polygyrus bakeri* in DEREG mice. Depletion of FoxP3^+^ Tregs was associated with increased type 2 cytokine responses, but no effect on worm burden was seen 134. The mode of action of the FoxP3^+^ Tregs was not investigated. Taken together, the available data so far present a mixed picture in terms of the role for FoxP3^+^ Tregs in GI nematode infection, and this needs to be resolved. It is of note that in other immune conditions, subsets of FoxP3^+^ Tregs have been identified such as the IL-33 driven ST2^+^ FoxP3^+^ Tregs that prolong transplant graft survival 143, and it may be that similar populations require examination in the context of GI nematode infection.

### The roles of TGF- β and IL-10

Much data, however, have accumulated to show that the cytokines IL-10 and TGF-β are key to FoxP3^+^ Treg effector function in a number of systems including intestinal inflammation 144. Moreover, it has been known that these cytokines play key roles in GI nematode infection. The data on the TGF-β mimic produced by *H. polygyrus bakeri* would argue that this cytokine has a key role to play 139. Data from this system also support a role for this molecule in limiting mucosal Th1 and Th2 cytokine production and enhancing IL-10 production 145.

Administration of neutralizing anti-TGF-β monoclonal antibody to chronically infected C57BL/6 mice given a low *T. muris* dose had little effect on parasite load or pathology although a small but significant depression of type 2 responses was identified (Lawrence and Grencis, unpublished observations). In contrast, it has been known for some time that IL-10 plays a key role in the *T. muris* system. Complete IL-10 null animals given a high-dose infection do not expel their infection and eventually develop pronounced colitis suggesting that IL-10 plays a role during both the generation of the protective type 2 response in addition to controlling IFN-γ-mediated intestinal inflammation 146. Moreover, using conditionally targeted mice, it was clear that the IL-10 critical to control of the *T. muris* associated inflammation is derived from the CD4^+^ T cell 147.

**Figure 3:**
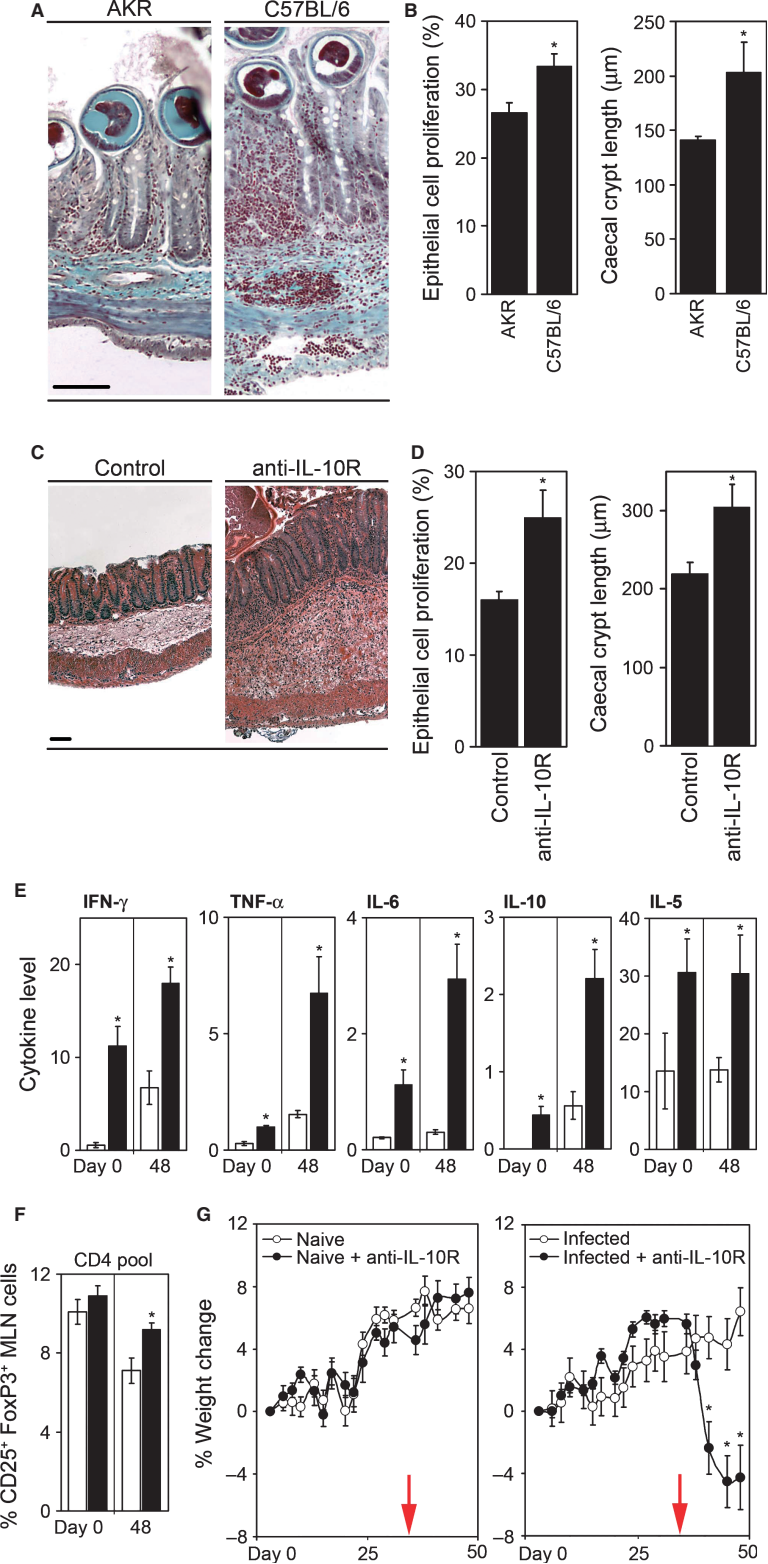
Cecal pathology associated with a chronic *Trichuris muris* infection is regulated via interleukin-10 (IL-10). AKR and C57BL/6 mice were orally infected with 25 embryonated *T. muris* eggs. Sectioned cecal tissue was taken at day 35 p.i., and stained using Gomori's one-step trichrome. Scale bar = 100 μm (A). Cecal crypt length was measured as an indicator of infection-associated pathology (B). Changes in IEC proliferation were assessed by quantifying the percentage of bromodeoxyuridine (BrdU)-labeled cells per crypt (B). To assess the role of IL-10, AKR mice were infected with approximately 100 *T. muris* eggs and treated with anti-IL-10R monoclonal antibody (mAb). Cecal tissue was sectioned, H&E stained (C), and crypt length measured (D). Intestinal epithelial proliferation was assessed using staining for BrdU uptake (D). MLN cells were isolated from anti-IL-10R mAb (closed bars) and control mice (open bars), restimulated with parasite Excretory/Secretory (ES) antigen for 24 h, and supernatant levels assayed for IFN-γ, TNF-α, IL-6, IL-10 (ng/ml), and IL-5 (pg/ml) by Cytokine bead array (Becton Dickinson) (E). Frequency of mesenteric lymph node (MLN) Treg cells was determined by flow cytometry. Data represent the percent of CD25^+^ FoxP3^+^ cells within the CD4^+^ T-cell pool (F). All mice were weighed and the percentage change determined (G). Values represent mean ± SE for five animals per group. * Significant difference between experimental groups (*P* < 0.05).

The fact that CD4^+^ T-cell-derived IL-10 is important in regulating both immunity and pathology following a high dose *T. muris* infection prompted further investigation of the role of IL-10 during low dose chronic infection in this system. There is a marked difference between different inbred strains of mouse in their ability to control the intestinal pathology observed following low-dose chronic *T. muris* infection, e.g. between AKR mice and the commonly used C57BL/6 strain. Histologically, C57BL/6 mice exhibit a greater cellular infiltrate penetrating the *lamina propria*, submucosa, and basal muscle layers (*Fig. *3*A*). This is also reflected by an enhanced crypt hyperplasia and increased epithelial proliferation (*Fig. *3*B*). Thus, unlike C57BL/6 mice, AKR mice appear to be able to control *T. muris*-induced intestinal pathology effectively. Treatment of high-dose infection in AKR mice (which will naturally progress to chronic infection) with anti-IL-10R monoclonal antibody only during the chronic phases of infection interfered with control of gross pathology (*Fig. *3*C*), crypt hyperplasia, and intestinal epithelial cell turnover (*Fig. *3*D*), a situation more reminiscent of the infected C57BL/6 strains. The anti-IL-10R antibody treatment was associated with significant elevations in a number of cytokines including IFN-γ, TNF-α, IL-6, and IL-10 (*Fig. *3*E*). These changes were also mirrored by an increase in an elevation in percentage of FoxP3^+^ Tregs in the MLN (*Fig. *3*F*). Overall, animal health following anti-IL-10R treatment was measured via assessment of body weight. The data clearly showed that following infection animals continued to put on weight until commencement of antibody treatment when a rapid weight loss ensued (*Fig. *3*G*). Similar experiments following low dose experiments in the C57BL/6 strain produced analogous data (Humphreys & Grencis, unpublished data). This data clearly show that long-lived *T. muris* infection represents a dynamic regulatory situation in which IL-10 plays a critical role once chronic infection has established. Interference with the action of IL-10 without removal of infection can lead to lethal consequences.

The critical source of the IL-10 is the CD4^+^ T cell, although which subpopulation has not been defined. FoxP3^+^ CD4^+^ IL-10^+^ T cells and FoxP3^−^ CD4^+^ IL-10^+^ T cells are present in the draining lymph node during chronic infection together with populations of IFN-γ^+^ CD4^+^ T cells and a small but significant population of IFN-γ^+^ IL-10^+^ CD4^+^ T cells (Steketee, Lawrence, and Grencis, unpublished observations). The relative importance of IL-10 derived from one T-cell subpopulation as opposed to another remains to be determined, but by extrapolation from studies in DEREG mice 113, FoxP3^+^ Tregs would be unlikely to be the major critical source.

The precise function of IL-10 in controlling the pathology during *T. muris* infection is also unknown. It is interesting to note that recent experimental work using non-infectious models of colitis have indicated that IFN-γ drives expression of the IL-10R1 in colonic epithelium and that IFN-γ and IL-10 upregulate the target gene SOCS3 in intestinal epithelium, which can help control inflammation 148. All four genes are known to be significantly upregulated during chronic *T. muris* infection 32,149.

The data collectively suggest that IL-10 plays a significant role during chronic *T. muris* infection. By extension to other GI nematode systems, the data are less clear but supportive in part. It is also entirely possible that the balance of regulatory cytokines such as IL-10 and TGF-β may change with host genetics, infection level, and stage of parasite life cycle and, therefore, in function.

**Figure 4:**
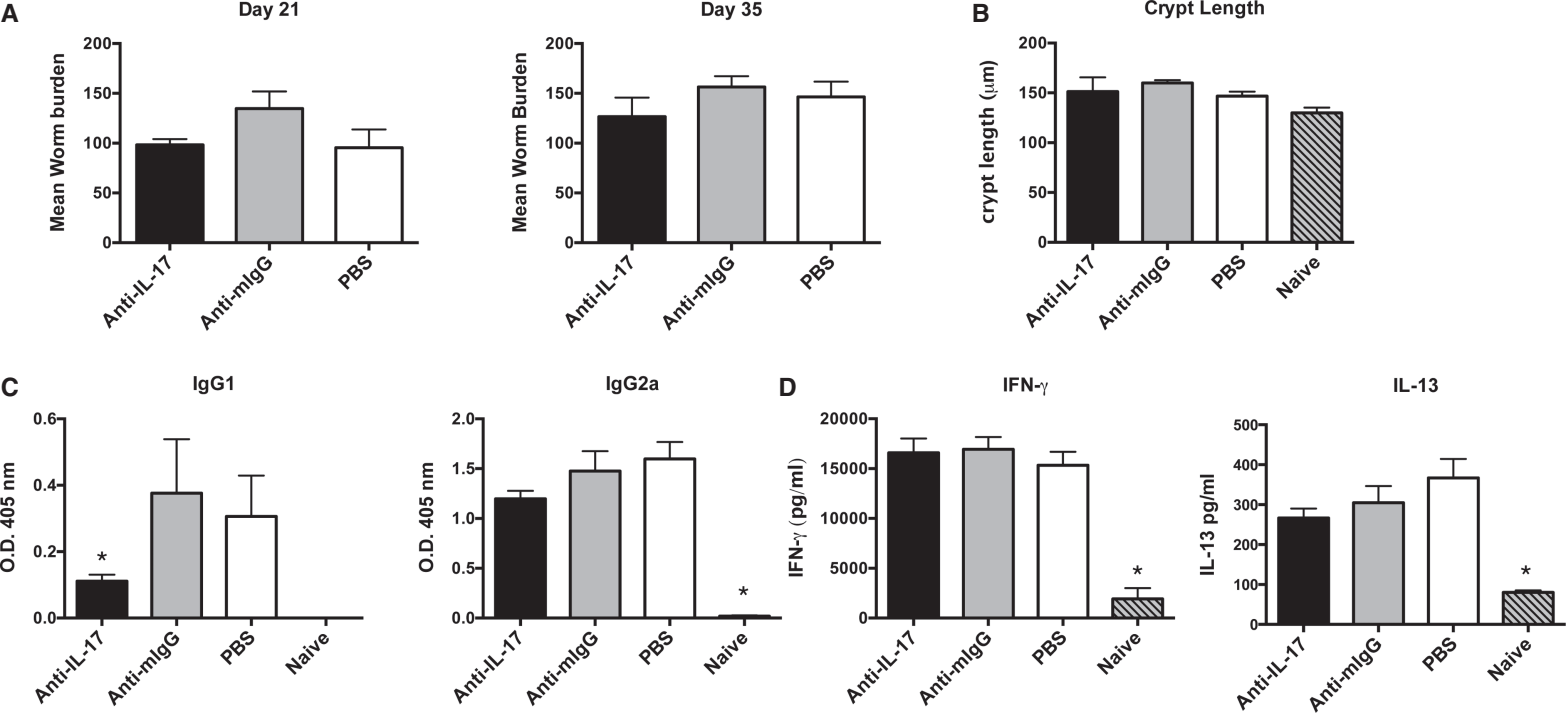
Neutralization of interleukin-17 (IL-17) has no effect on *Trichuris muris* worm burden and immune responses following chronic infection. Groups of five AKR mice were treated with 0.5 mg of anti-IL17, mouse IgG (a kind gift from J. Van Snick), or PBS intraperitoneally every 3–4 days throughout infection from day 0. (A) Mean worm burden at days 21 and 35 postinfection (p.i). (B) Caecal crypt length. (C) Parasite-specific IgG1 and IgG2a were measured by ELISA using *T. muris* excretory secretory (ES) as antigen and day 35 postinfection serum at a dilution of 1/80. Values represent mean ± SE. * Significant difference between experimental groups (*P* < 0.05). (D) Cytokines were measured in supernatants of mesenteric lymph node cells (MLNC) restimulated *in vitro* with 50 μg/ml of *T. muris* ES for 24 h. Cytokine levels were evaluated by Cytokine bead array (Becton Dickinson).

The role of IL-17A has received considerable attention in terms driving inflammation and protective immunity at both mucosal and non-mucosal sites 150–152. Moreover, the role of bona fide Th17 cells has been an intensive area of research. With regards to GI nematode infections, a critical role for either cytokine or Th subset has yet to be convincingly demonstrated. This is perhaps surprising, as elevated expression of genes in the Th17 pathway is often evident following many experimental GI nematode studies and IL-17 production is a feature of *in vitro* recall cytokine assays following infection. In the *T. muris* system, for example, where the data would indicate IL-17A is associated with chronic infection from both gene expression 149 and cytokine responses *in vitro*, *in vivo* neutralization using anti-IL-17 monoclonal antibodies has so far failed to demonstrate a major role during chronic infection (*Fig*.4). The studies of Fasnacht *et al*. 147 using the IL-10/gp130 CD4^+^ T-cell conditional knockout mouse in conjunction with high level *T. muris* infection did support a role for IL-17A (though not T-cell derived), but only in the absence of IL-10. This is unlikely to happen during *T. muris* infection in WT mice.

## Consequences of nematode induced immunoregulation in the GI tract

**Figure 5:**
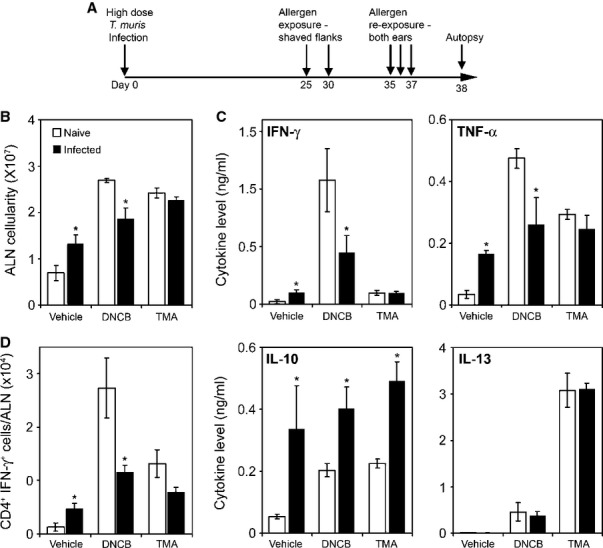
A chronic *Trichuris muris* infection preferentially regulates responses to a Th1 polarizing skin allergen. Naive and *T. muris*-infected AKR mice were repeatedly exposed topically to either the Th2-polarizing allergen Trimellitic anhydride (TMA), the Th1-polarizing allergen 2,4-dinitrochlorobenzene (DNCB), or vehicle (A). Auricular lymph nodes (ALN) draining both ears were isolated, and the mean cellularity was calculated (B). These cells were cultured and supernatant levels of IFN-γ, TNF-α, interleukin-10 (IL-10), and IL-13 were measured by cytokine bead array (Becton Dickinson) (C). Frequency of CD4^+^ IFN-γ^+^ ALN cells was determined by FACS analysis. Data represent the number of cytokine-positive cells per ALN (D). Values represent mean ± SE for four animals per group. * Significant difference between experimental groups (*P* < 0.05).

**Figure 6:**
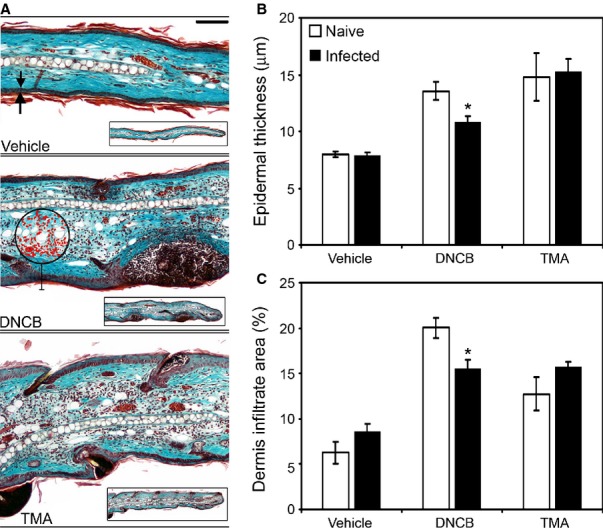
Ear pathology induced by a Th1-polarizing skin allergen is regulated by a chronic *Trichuris muris* infection. Naive and *T. muris*-infected AKR mice were exposed to either the Th2-polarizing allergen Trimellitic anhydride (TMA), the Th1-polarizing allergen 2,4-dinitrochlorobenzene (DNCB), or vehicle. Whole ears were sectioned and stained using Gomori's one-step trichrome stain. Scale bar = 50 μm, arrows indicate epidermal thickness measured, circle and highlighted cells (red) indicate a software-defined area used to quantify the dermal cellular inflammatory infiltrate (A). Epidermal thickness (B) and the dermal inflammatory infiltrate (C) were measured as an indicator of induced allergen-associated pathology. Values represent mean ± SE for four animals per group. * Significant difference between experimental groups (*P* < 0.05).

**Figure 7:**
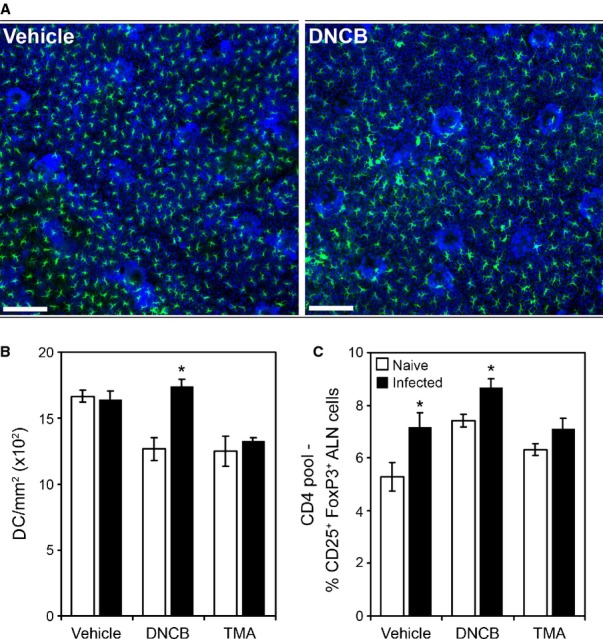
Dendritic cell migration induced by a Th1-polarizing skin allergen is prevented by a chronic *Trichuris muris* infection. Naive and *T. muris*-infected AKR mice were exposed to either the Th2-polarizing allergen Trimellitic anhydride (TMA), the Th1-polarizing allergen 2,4-dinitrochlorobenzene (DNCB), or vehicle. Ear dorsal epidermal sheets were prepared, stained for dendritic cells (DC) using anti-MHC class-II IA^K^ and for cell nuclei using DAPI. Scale bar = 50 μm (A). As an indicator of allergen-associated induction of DC migration, the epidermal density of MHC class-II^+^ was ascertained for each animal (B). Auricular lymph nodes (ALN) Treg cell frequency was determined by FACS. Data represent the percentage of CD25^+^ FoxP3^+^ cells within the CD4^+^ pool (C). Values represent mean ± SE for four animals per group. * Significant difference between experimental groups (*P* < 0.05).

The clear demonstration that immunoregulation is a major component of GI nematode infection has consequences in terms of effects for host physiology, particularly with regards to how the host deals with concurrent antigenic, infectious, and inflammatory challenge. This is of considerable fundamental and translational interest. While not a major focus of this review, it is pertinent to highlight some work in this area as illustrative of the considerable influence GI nematodes have. There are numerous studies both descriptive and mechanistic that show that GI nematodes can influence the response to antigenic challenge (and co-infection) both within mucosal and non-mucosal sites. The efficacy of vaccines and co-infections in humans is well documented and broadly suggests that downregulation of both humoral and cell mediated immunity is often a feature observed in individuals already carrying a GI nematode infection compared to nematode-free individuals 153–155. In the laboratory, using models of chemical contact hypersensitivity that depend upon Type 1 or type 2 responses 156,157, it can be seen that a chronic *T. muris* infection modulates challenge responses to the chemical sensitizers in the ear, a remote non-mucosal site distant from the cecum. Moreover, modulation is apparent only against type 1 cytokine sensitization and not those chemicals that activate the type 2 cytokine or Th2 pathway changing both cytokine responses (*Fig. *5) and ear pathology (*Fig. *6). This depression in sensitization was associated with a reduction in movement of class-II positive cutaneous DCs cells from the skin and elevated IL-10 levels in the ear draining lymph node (*Fig. *7). Local IL-10 delivery is known to inhibit movement of cutaneous DCs from the ear in these sensitization models 156. This would suggest that the immunoregulatory circuits induced by *T. muris* systemically are those associated with the immunity underlying chronic *T. muris* intestinal infection, i.e. a regulated Th1 response.

**Figure 8:**
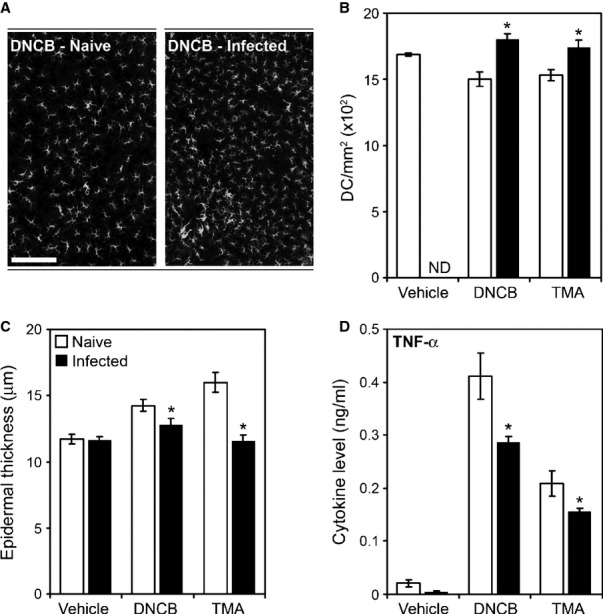
Dendritic cell migration, ear pathology, and TNF-α production associated with skin allergen exposure are regulated by a *Heligmosomoides polygyrus* bakeri infection. Naive and *H. polygyrus bakeri*-infected BALB/c mice were exposed to either the Th2-polarizing allergen Trimellitic anhydride (TMA), the Th1-polarizing allergen 2,4-dinitrochlorobenzene (DNCB), or vehicle. Whole ears were sectioned and stained to visualize tissue structure and allergen-associated epidermal thickening was measured (A). As an indicator of allergen-induced dendritic cells (DC) migration, the ear epidermal density of MHC class-II^+^ cells was ascertained, quantified (B), and visualized (C). ND = not done, scale bar = 50 μm. ALN cells were isolated and cultured, and supernatant levels of TNF-α were measured by Cytokine bead array (Becton Dickinson) (D). Values represent mean ± SE for four animals per group. * Significant difference between experimental groups (*P* < 0.05).

Similar experiments following chronic infection with *H. polygyrus bakeri* did not show this dichotomy with ear responses downregulated to both type 1 and type 2 chemical sensitizers (*Fig. *8). Chronic infection with *H. polygyrus bakeri* is not associated with a robust IFN-γ response like *T. muris*. The effects of GI nematode infection have also been demonstrated for non-antigenic inflammatory diseases. For example, chronic *T. muris* infection has a dramatic influence on cerebral ischemia. Using models of experimental stroke in mice chronic infection increases the severity of stroke and this has been shown to be dependent on the action of CCL5 expression in the brain. This is not evident following acute infection by *T. muris* 158,159. These data underpin the concept that infection by gut dwelling nematodes is far from silent 60, and the prevalence of such infections indicates that studies of individuals where infection is endemic cannot simply assume that the ongoing GI nematode infection does not have an effect from immunological aspects through inflammation and even drug accessibility via the intestinal tract.

## The GI tract: not just worms alone

The gastrointestinal tract is also one of the major sites of the body's microbial communities, particularly the cecum and colon. The evidence that the intestinal microbiome plays a pivotal role in the immune response in the gut and beyond to overall health is incontrovertible with numerous studies demonstrating a key functional role of certain bacterial species in stimulating and modulating immunoregulatory processes. The work generated in experimental laboratory systems and principles established are now reflected in numerous human studies 160–165.

This is of clear interest to studies of immune responses to GI nematodes. There is the additional realization that the evolution of the immune system has gone hand in hand with the evolution of the intestinal microflora and, importantly, in the presence of constant GI nematode infection 166,167. There is growing evidence from a number of experimental systems that infection by gut nematodes changes the resident intestinal microflora 168,169. Studies began before the advent of molecular approaches 170–172, but have expanded significantly in the wake of techniques that can identify species of bacteria regardless of cultivability. Following *H. polygyrus bakeri* infection, studies have shown changes in gut microbiota of the terminal ileum, cecum, and colon, even though the parasite occupies the small intestine. Common changes observed are a significantly increased abundance of members of the *Lactobaillaceae* 173,174. Studies also identified significant increases in the *Enterobacteria* in all gut compartments following infection 174, which has been seen in a number of inflamed gut systems 175,176. Experimental studies in pigs following a large dose of *T. suis* (20 000 eggs) have demonstrated considerable change in colonic microflora and differences between animals that retain some worms as opposed to those that expel their parasites. Associated changes were seen in bacterial groups that were involved in digesting fiber 56,177. It is already known that *T. suis* infection is associated with increased susceptibility to *Campylobacter* infection in pigs 178 although functional relationships between the microflora, worm infection, and other pathogens remain to be defined.

Data from studies in human GI nematode infections using fecal samples to assess the microflora are relatively scarce but where available have not shown major influences of gut worms although there are indications that co-infections with multiple GI nematode infections (which are very common) may influence microbiome. Populations of children infected with *T. trichiura* and/or *Ascaris lumbricoides* in Ecuador did not find major changes in gut flora with either infection alone, although there was some indication of effects when individuals had both infections 179. Clearly, studies in the field differ dramatically from those carried out under laboratory conditions, and further work is necessary.

The close biological relationship between the host immune system, the gut dwelling nematode, and the microflora is exemplified by the *T. muris* mouse system in that the cecal microflora induces parasite hatching at the preferred site of infection, the cecum. *In vitro* it can be shown that bacterial species can induce hatching by binding to the polar plugs of the *T. muris* eggs, the sites where the larval parasites emerge. Treatment of mice with antibiotics at the time of infection not only depresses the hatching of parasite eggs, but also alters the host immune response to the worms that do manage to establish. When microfloral numbers are depressed, the type 2 cytokine response to *T. muris* is elevated and worms are expelled more quickly 180. Interestingly, TLR4 (and MyD88)-deficient animals, i.e. those that do not respond well to PAMPs via signaling through common PRR (e.g. from bacterial species), also make enhanced Type 2 cytokine responses upon *T. muris* infections, and chronic infections are very difficult to establish 85. This raises the possibility that recognition of bacterial species via this route could indeed influence subsequent responses. It is noteworthy that anti-microbial products such as angiogenin 4 and cryptidins are upregulated following *T. muris* infection 181.

### Fewer myths and more mechanisms?

The above discussion leads to the conclusion that immunity to GI nematodes is a mixture of both resistance and susceptibility, usually concurrent and far from sterile. Experimental studies using well-defined models have identified clear mechanisms of resistance, dominated by IL-13 controlled responses that manifest themselves within the mucosal tissue and the secreted mucus gel. Model systems have also led to the identification of ILC2s and their induction and potency in driving protective effector mechanisms. Their interrelationship with CD4^+^ T cells is emerging as part of a co-ordinated defense strategy. It is also apparent that regulation is a necessary feature of long-lived GI nematode infection where multiple cell sources release regulatory cytokines.

These studies and others emphasize the importance of considering the mucosal immune response to infection *in toto* and not in isolation from other components that will have influenced the evolution of the immunity we see in the present day. A key area with regards to gut immunity and infection that deserves more attention is the role of diet and metabolism. The interactions between ILC populations and fat tissue, the major influence that diet has on microflora, and the interplay between bacteria, the products they secrete, and the host immune system, will influence and be influenced by GI nematodes. It can easily be envisaged how the resulting complex intestinal metabolome will impact on the host immunoregulatory network. Reductionist studies are critical to define mechanisms and construct hypotheses, but ultimately systems need to be considered and analyzed to gain real insight to the *in vivo* situation.
